# Silica precipitation potentially controls earthquake recurrence in seismogenic zones

**DOI:** 10.1038/s41598-017-13597-5

**Published:** 2017-10-17

**Authors:** Hanae Saishu, Atsushi Okamoto, Makoto Otsubo

**Affiliations:** 1 Renewable Energy Research Center, National Institute of Advanced Industrial Science and Technology (AIST), 2-2-9 Machiikedai, Koriyama, Fukushima, 963-0298 Japan; 2 Geological Survey of Japan, National Institute of Advanced Industrial Science and Technology (AIST), Tsukuba Central 7, 1-1-1 Higashi, Tsukuba, Ibaraki, 305-8567 Japan; 3 Graduate School of Environmental Studies, Tohoku University, 6-6-20 Aramaki-Aza-Aoba, Aoba-ku, Sendai, Miyagi 980-8579 Japan

## Abstract

Silica precipitation is assumed to play a significant role in post-earthquake recovery of the mechanical and hydrological properties of seismogenic zones. However, the relationship between the widespread quartz veins around seismogenic zones and earthquake recurrence is poorly understood. Here we propose a novel model of quartz vein formation associated with fluid advection from host rocks and silica precipitation in a crack, in order to quantify the timescale of crack sealing. When applied to sets of extensional quartz veins around the Nobeoka Thrust of SW Japan, an ancient seismogenic splay fault, our model indicates that a fluid pressure drop of 10–25 MPa facilitates the formation of typical extensional quartz veins over a period of 6.6 × 10^0^–5.6 × 10^1^ years, and that 89%–100% of porosity is recovered within ~3 × 10^2^ years. The former and latter sealing timescales correspond to the extensional stress period (~3 × 10^1^ years) and the recurrence interval of megaearthquakes in the Nankai Trough (~3 × 10^2^ years), respectively. We therefore suggest that silica precipitation in the accretionary wedge controls the recurrence interval of large earthquakes in subduction zones.

## Introduction

Earthquake recurrence intervals are commonly used to predict the timescales of future earthquakes^1–3^. However, many unresolved factors add ambiguity to any discussion of the timescales of seismic cycles, including which parameters control earthquake triggering and the timescales required to change crustal conditions from aseismic to seismic. It has been suggested that the distribution of high pore pressures within the seismogenic zone controls earthquake occurrence, as inferred from a combination of seismic data, the porosity of *in situ* rocks, and numerical simulations^4–7^. The interseismic period related to the cycling of fluid pressure has been explained by changes in tectonic stress and fault strength^8^. In particular, the buildup and release of pore fluid pressure during a seismic cycle promote temporal changes in fault strength. The dissolution and precipitation of minerals play an important role in closing dilatational fractures generated during the co-seismic period and reducing the porosity of rocks^9^. However, the evolution of relevant hydrological properties (e.g., pore fluid pressure, porosity, and permeability) related to water–rock interaction during the interseismic period is poorly understood.

Silica is a dominant component of crustal rocks, and widespread quartz veins in subduction zones at seismogenic depths are taken as proof of significant fluid flow and silica precipitation^10–12^. Silica–water interaction may play a crucial role in a wide range of seismic phenomena; e.g., the rapid precipitation of large amounts of silica due to a large pressure drop in response to an earthquake^11,13^, fault weakening by silica gel formation on fault surfaces^14,15^, and pressure solution and diffusion of silica that occur in relation to slow slip earthquakes^12,16^. In particular, silica precipitation effectively seals cracks and faults, and could therefore significantly influence the buildup of fluid pressure during the interseismic period^8,17,18^. However, no studies have quantitatively discussed the timescales of quartz vein formation and earthquake recurrence intervals within subduction zones.

Here we propose a novel kinetic model of quartz vein formation that allows us to estimate the sealing times of isolated cracks based on pore-fluid pressure drop within the crack, advective flow of silica-rich fluids from the peripheral host rock, and quartz crystal growth in the seismic zone. Our model quantifies the relationship between the recurrence intervals of earthquakes and the sealing timescales of quartz-filled extensional cracks around the Nobeoka Thrust, southeastern Japan, a typical seismogenic megasplay fault in an ancient subduction zone.

## Geological setting and occurrence of extensional quartz veins

**Figure 1:**
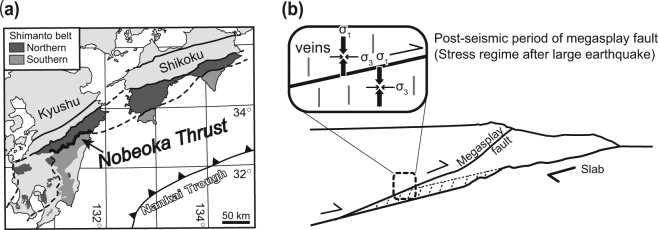
Geological conditions and the formation of extensional veins associated with thrust. (**a**) Geological setting of the Nobeoka Thrust, southwest Japan (modified after ref.^52^). (**b**) Schematic model of spatial change of stress regimes at post-earthquakes and formation of extension cracks along the subduction zone (modified after ref.^21^). The map (**a**) was created by using Adobe Illustrator^53^ version CS6 software.

The Nobeoka Thrust is a major fault that bounds the northern and southern Shimanto belts of Kyushu, southwestern Japan^19^ (Fig. 1a), traceable for >800 km in the Cretaceous–Neogene Shimanto Belt accretionary complex parallel to the modern Nankai Trough^19^. The thrust is considered a fossilized megasplay fault due to the presence of pseudotachylite in its hanging wall damage zone^20^ and its paleotemperatures^19^. Its total estimated displacement of ~8.6–14.4 km, based on a 70 °C temperature difference between the hanging wall and footwall, is comparable to the deeper part of the modern megasplay fault in the Nankai Trough^19,21^.

The veins around the Nobeoka Thrust are classified into three types: extensional crack-filling veins (mode I cracks), fault-filling veins, and post-mélange veins^19,21–23^. The extensional crack-filling veins, which cut phyllitic shale, mélange, cataclasite, and composite planer fabrics, are abundant in both the hanging wall and footwall of the Nobeoka Thrust^21^. The stress inversion reveals that the extensional quartz veins formed under a normal faulting type stress regime, with the orientation of the minimum principal stress (*σ*_3_) axis being almost horizontal and trending roughly NNW−SSE in both the hanging wall and footwall^21^ (Fig. 1b). The negative stress of the reverse faulting type stress regime, combined with the *σ*_3_ axis subparallel to the slip direction of the Nobeoka Thrust (top to the SSE)^21,24^, indicates that the normal faulting type stress regime at the time of fracture opening was secondary stress generated by slip of the Nobeoka Thrust^21^. The temporal stress changes are similar to those of Mw 9.0 2011 Tohoku-Oki earthquake^25^ and to large trench-type earthquakes along the décollement^26^. The microstructural features of syntectonic mineral veins have been interpreted as evidence of temporal fluctuations in fluid pressure during repeated earthquake cycles within a seismogenic megasplay fault in an ancient subduction zone^22^. The extensional veins are consistent with multiple episodes of slip on the Nobeoka Thrust. Analyses of fluid inclusions hosted by rocks of the Shimanto belt in Kyushu reveal that the fluid salinity is similar to or lower than that of seawater^27^. Geochemical and mineralogical analyses suggest that extensional veins around the Nobeoka Thrust formed from relatively oxidized, locally derived pore fluids with neutral pH^22^. Analyses of vitrinite reflectance and fluid inclusions reveal that vein formation occurs under both high *P–T* conditions (260–340 °C, 235–250 MPa) and low *P–T* conditions (140–250 °C and 150–190 MPa) in the hanging wall and footwall of the Nobeoka Thrust^19,28,29^. The difference in temperature across the fault was generated by activation of the Nobeoka Thrust as an out-of-sequence-thrust or megasplay fault^19^. The normalized pore fluid pressure ratio, the lower bound of the maximum fluid pressure level^30^, is ~0.95 for a tensile strength of 10 MPa^21^. This stress condition means that the tensile overpressure exceeds *σ*_3_ during the period of vein formation. The formation of high-angle extensional fractures occurs only when tensile overpressure *P*_f_ > *σ*_3_ is attained^31^.

Great earthquakes along the Nankai Trough occurred at intervals of 9 × 10^1^–3 × 10^2^ years in the period covered by historical records (684–1946 A.D.)^2^. The high pore pressures were expected to be close to lithostatic pressure along the mega-splay fault of the Nankai Through^7^. However, there is no consensus on the recurrence intervals of earthquakes on splay/megasplay faults around the Nobeoka Thrust or those of megathrust earthquakes along the Nankai Trough. If we treat the postseismic period (i.e. the period under the secondary stress regime) as having effectively zero aftershock activity after roughly one-tenth of the recurrence interval (as inferred from compiled data and numerical estimates of mainshock recurrence times, and observed and predicted aftershock sequence durations^1^), the timescale required to open extensional cracks in the Shimanto accretionary wedge is 9 × 10^0^–3 × 10^1^ years.

**Figure 2:**
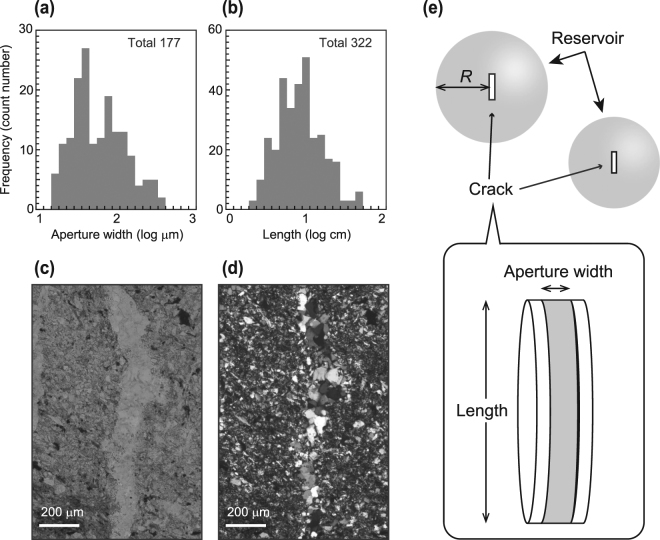
Photos and schematic image of extensional quartz veins observed along the Nobeoka Thrust. The log-size histogram of quartz veins of aperture width measured by using the thin section samples of quartz veins (**a**), and length measured at the outcrop of the Nobeoka Thrust (**b**). The photographs of (**c**) open-nichol and (**d**) cross nichol of a thin section of quartz veins around the Nobeoka Thrust. (**e**) Schematic of a crack in a reservoir that formed via advection and kinetic reaction (Supplementary Method).

We observed discrete extensional veins in the hanging wall of the Nobeoka Thrust, filled mainly with quartz and lesser calcite. The aperture widths (*w*_v_) and lengths (*l*_v_) of quartz veins were estimated from eight thin sections (48 × 22 mm) and field measurements, respectively; both measurements are log-normally distributed, with *w*_v_ = 1.3 × 10^1^–3.4 × 10^2^ μm (geometric mean 5.2 × 10^1^ μm; Fig. 2a) and *l*_v_ = 1.9 × 10^0^–5.0 × 10^1^ cm (geometric mean 7.4 × 10^0^ cm), with a few veins being longer than 5.0 × 10^1^ cm (Fig. 2b). The vein quartz grew on quartz grain surfaces in vein walls (Figs. 2c,d), and there is no evidence of repeated crack–seal events (i.e. inclusion bands^32^).

## Model of quartz vein formation

We propose a model of quartz vein formation associated with fluid advection from host rocks and silica precipitation in a crack (Supplementary Method). An isolated disk-shaped crack exists at the center of a spherical host-rock reservoir of radius *R* meters (Fig. 2e). Pore fluids in the host rock are assumed to be always saturated with quartz at constant temperature and lithostatic pressure due to the pressure dissolution or free surface dissolution of sandstone^22^. At the time of an earthquake, the crack is opened, which causes a fluid pressure drop in the crack. The difference in fluid pressure between the host rock and the crack (Δ*P* = *P*_host_ − *P*_crack_) induces fluid advection from the host rock into the crack and silica precipitation in the crack. The two sides of the circular crack walls are the input paths of fluid from the host rock into the crack, whereas fluid flows radially through the crack for a distance equal to the radius. Based on the quartz vein textures (Figs. 2c,d), we consider silica precipitation as quartz overgrowth on pre-existing surfaces, governed by the kinetic equation of quartz overgrowth^33^. We can define two kinds of fluid volume associated with quartz vein formation: (1) the fluid volume required for sufficient silica precipitation to seal a crack completely, which depends on the pressure drop and the kinetics of silica precipitation and (2) the fluid volume of the reservoir that supplies SiO_2_-supersaturated fluid by advection, which follows Darcy’s law. We solve for the unique sealing time (*t*_s_) simultaneously by iterating with the constraint that the two fluid volumes are equal (Supplementary Method).

**Figure 3:**
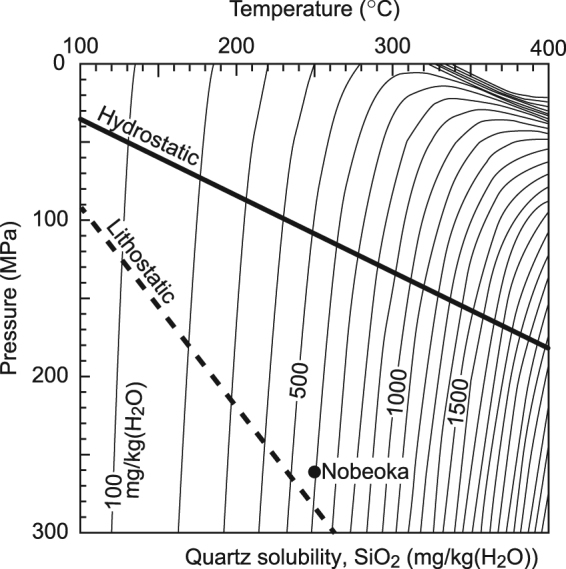
*P*-*T* diagram of quartz solubility with the conditions of the Nobeoka Thrust. Quartz solubility (SiO_2_) is 6.0 × 10^2^ mg/kg(H_2_O) at a temperature of 250 °C and a fluid pressure of 260 MPa in the host rock along the Nobeoka Thrust (black circle). Quartz solubility gradients are indicated by thin solid lines in units of mg/kg(H_2_O). The fluctuation between lithostatic (broken bold line) and hydrostatic (solid bold line) fluid pressure in the model of this study occurs at a temperature gradient of 20 °C/km.

In examining the formation of extensional cracks, we consider the realistic conditions around the Nobeoka Thrust (Supplementary Note). The initial pore fluid pressure and temperature of the extensional crack are set to 260 MPa and 250 °C, respectively, which are equivalent to the lithostatic conditions at a depth of 10 km on the Nobeoka Thrust (Fig. 3). The vein-forming fluids around the Nobeoka Thrust are considered to have neutral pH^22^ and low salinity^27^, and we assume a constant SiO_2_ concentration in the fluid in the host rock (*C*_SiO2_ = 6.0 × 10^2^ mg/kg(H_2_O)), which is taken from the solubility of quartz in pure water at 250 °C and 260 MPa (Fig. 3)^34^. The SiO_2_ concentration in the fluid in the host rock (*C*_SiO2_) is 6.0 × 10^2^ mg/kg(H_2_O) as a result of pure water saturated via quartz at 250 °C and 260 MPa (Fig. 3)^34^. As a typical example, we consider a crack with aperture width *w*_v_ = 5.2 × 10^1^ μm and length *l*_v_ = 7.4 × 10^0^ cm (diameter), which are the geometric mean values of each parameter for extensional veins along the Nobeoka Thrust (Figs. 2a,b). We use a host-rock permeability of 1.0 × 10^−19^ m^2^, which is the average of previous measurements using a triaxial pressure apparatus (1 × 10^−20^ to 1 × 10^−18^ m^2^)^35^. The porosity of the host rock ranges from 3% to 10%, as measured by geophysical wireline logs across the Nobeoka Thrust^23^. In this study, we use a minimum porosity of 3% for most of the calculations, and we show that porosity has a minor influence on estimates of sealing time. The porosity and permeability of the host rock are set to be constant during crack sealing because the volume of quartz required to seal a crack is much smaller than that of the fluid reservoir in the host rock.

## Results

**Figure 4:**
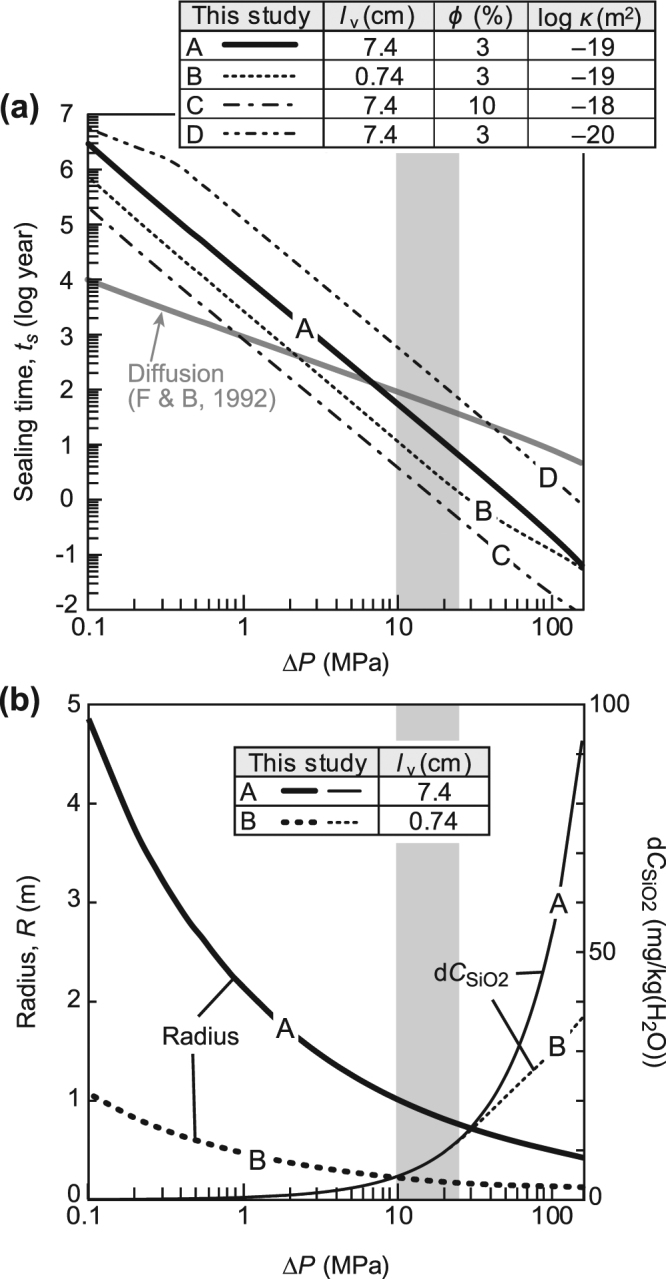
Crack sealing time and conditions. (**a**) Sealing times of extensional quartz veins calculated using our model (black lines, A–D) and a diffusion model (grey line)^37^. (**b**) Reservoir radius (bold lines) and differences in fluid SiO_2_ concentrations (thin lines). Fluid pressure drop (Δ*P*) is from 0.1 to 160 MPa. ‘A’ (solid black line) is a basic model comprising a geometric mean crack of aperture width (*w*_v_ = 5.2 × 10^1^ μm) and length (*l*_v_ = 7.4 × 10^0^ cm) in a host rock of porosity (*ϕ = *3%) and permeability (*κ = *1 × 10^–19^ m^2^). ‘B’ (dotted line) is a model comprising the reference size of a logarithmic–non-linear function (*l*_v_ = 7.4 × 10^–1^ cm). The sealing time of a geometric mean size crack depending on porosity and permeability, is in the range between C (*ϕ = *10%, *κ = *1 × 10^–18^ m^2^) (chain line) and D (*ϕ = *3%, *κ = *1 × 10^–20^ m^2^) (double-dot chain line). Grey zone indicates the realistic range of fluid pressure drops along the Nobeoka Thrust (Δ*P* = 10–25 MPa).

When increasing Δ*P* from 0.1 to 160 MPa, so that the pore fluid pressure in the crack decreases from lithostatic to hydrostatic (Fig. 3), the degree of supersaturation in the crack (*C*_SiO2_/*C*_SiO2,Qtz,eq_ − 1) increases from 7.1 × 10^−5^ to 1.9 × 10^−1^, and the time taken for complete sealing of a crack becomes much shorter: for a typical crack, with *w*_v_ = 5.2 × 10^1^ μm and *l*_v_ = 7.4 × 10^0^ cm, the time decreases from 3.1 × 10^6^ years (3.1 My) to 7.3 × 10^−2^ years (27 days) (Fig. 4a), where the reservoir size falls into the radius range *R* = 4.9 × 10^0^ to 3.9 × 10^−1^ m (Fig. 4b). While passing through the crack, the silica concentration (*C*_SiO2_) decreases to a level close to the solubility of quartz at each *P*_crack_. Accordingly, the difference in SiO_2_ concentration between the input and output fluid, which corresponds to the amount of silica precipitation, increases from 4.2 × 10^−2^ mg/kg(H_2_O) at Δ*P* = 0.1 MPa to 9.4 × 10^1^ mg/kg(H_2_O) at Δ*P* = 160 MPa (Fig. 4b). In this Δ*P* range (0.1–160 MPa) at *T* = 250 °C, the sealing time (*t*_y_) in years can be expressed as a logarithmic–linear function:

1 $$\mathrm{log}\,{t}_{y}=-2.4\,\mathrm{log}\,{\rm{\Delta }}P+\mathrm{4.1.}$$

This relationship shifts in parallel with any change in crack size except under certain conditions; e.g., when the sealing time of a crack of one-tenth the geometric mean length (i.e., *l*_v_ = 7.4 × 10^–1^ cm) becomes similar to that of a mean-length crack with increasing Δ*P* (Fig. 4a). In a smaller crack, the residence time of fluid in the crack is too short to precipitate all of the supersaturated SiO_2_. Thus, the difference in SiO_2_ concentration between the input and output fluids in a smaller crack is smaller than that in a mean-sized crack at higher Δ*P* (Fig. 4b).

Given the mechanical properties of subduction megathrusts and the orientations of micro-fault systems (quartz veins), fluid pressure generated by extensional cracks can drop by up to 10% of lithostatic pressure (Δ*P* < ~25 MPa) during an earthquake^36^. In addition, assuming a rock tensile strength of 10 MPa, cracks remain open in the range Δ*P* = 0–10 MPa. Hence, an appropriate range of Δ*P* for the formation of extensional quartz veins at around the Nobeoka Thrust is 10–25 MPa, for which the sealing time of a geometric mean size crack is 6.6 × 10^0^–5.6 × 10^1^ years (Fig. 4a). In this case, the radius of a spherical reservoir is 7.5 × 10^–1^–1.0 × 10^0^ m (Fig. 4b). The sealing time depends on the conditions of the crack and the host rock. The sealing time of a crack of one-tenth the geometric mean length (i.e., *l*_v_ = 7.4 × 10^–1^ cm) ranges from 1.4 × 10^0^ to 1.2 × 10^1^ years at Δ*P* = 10–25 MPa (Fig. 4a). In the case of higher porosity (10%) and permeability (1 × 10^–18^ m^2^), the sealing time of a geometric mean size crack is 4.4 × 10^–1^–3.8 × 10^0^ years, while in the case of lower porosity (3%) and permeability (1 × 10^–20^ m^2^) the sealing time is between 6.6 × 10^1^ years and 5.6 × 10^2^ years at Δ*P* = 10–25 MPa (Fig. 4a).

In addition to advection, diffusion could contribute to silica transport during vein formation. We calculated the sealing time for a model of vein growth via diffusive silica transport in pore fluids at Δ*P* = 0.1–160 MPa, following ref.^37^ (Fig. 4a) (Supplementary Method). The driving force of silica transport in the diffusion model is the difference between the quartz solubility of the host rock and that of the cracks, and parameters such as Δ*P* are set to the same values as in the model of this study. The sealing time of a crack by diffusive transport of silica is slower than the time estimated for our model under higher Δ*P* (Fig. 4a), but faster than our model under lower Δ*P* (<8 MPa). In the possible range of fluid pressure drops along the Nobeoka Thrust (Δ*P* = 10–25 MPa), the sealing time in the model of this study is 10^1^–10^2^ times faster than in the diffusive model (Fig. 4a). This implies that fluid advection is the dominant transport mechanism during extensional quartz vein formation along the Nobeoka Thrust, even if both processes occur there.

**Figure 5:**
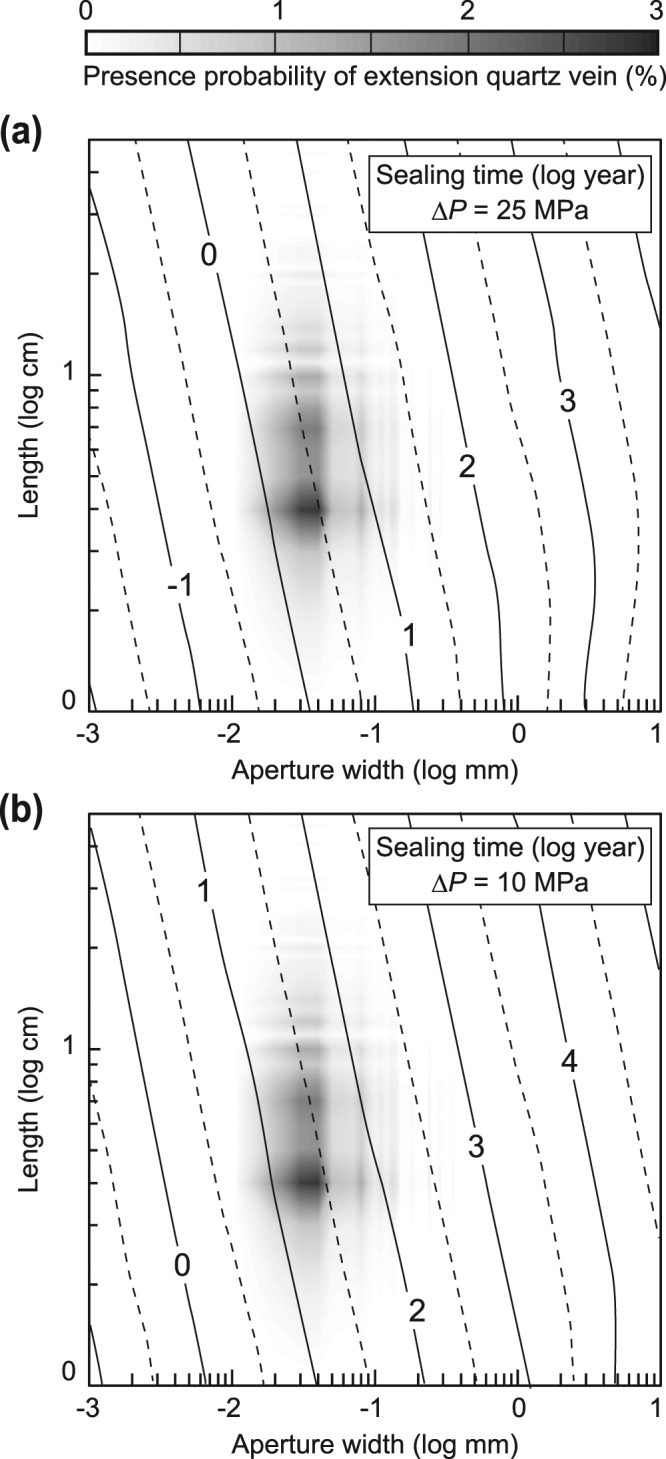
Probable sealing time of extensional quartz veins along the Nobeoka Thrust as a function of length and width of a crack. The difference in fluid pressure between the host rock and the crack (Δ*P*) is 25 MPa (**a**) and 10 MPa (**b**).

Crack volume is one of the critical factors for controlling the sealing time (Fig. 5). At Δ*P* = 25 MPa, doubling the crack aperture or crack length increases the sealing time by a factor of two or four, respectively. We roughly estimated the presence probability of each volume of vein (0%–3%) by 225 all possible combinations of aperture width (Fig. 2a) and length (Fig. 2b). Figure 5 shows that most extensional veins in the Nobeoka Thrust seal within a period on the order of 1 × 10^0^–1 × 10^1^ years at Δ*P* = 25 MPa (Fig. 5a) and on the order of 1 × 10^1^–1 × 10^2^ years at Δ*P* = 10 MPa (Fig. 5b). Thus, in the expected range of Δ*P* = 10–25 MPa, extensional cracks along the Nobeoka Thrust are sealed at timescales on the order of 1 × 10^0^–1 × 10^2^ years.

**Figure 6:**
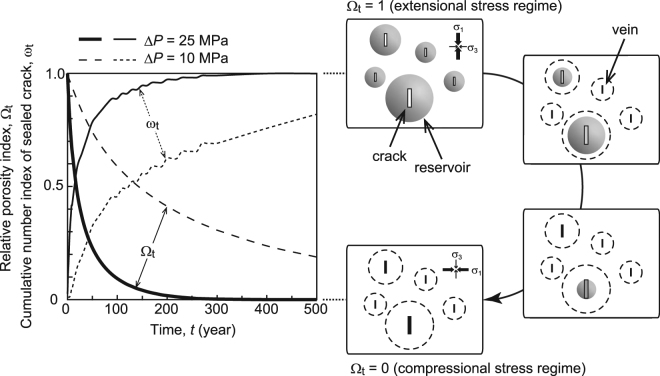
Temporal evaluation of relative porosity index and cumulative number index of sealed crack with conceptual image of crack sealing process. Relative porosity index (*Ω*_t_) and cumulative number index of sealed crack (*ω*_t_) at Δ*P* = 25 MPa (solid lines) and 10 MPa (broken lines).

We clarify the effects of silica precipitation on the evolution of hydrological properties of an accretionary wedge. Because it is impossible to know the exact number of cracks generated during a single earthquake, we define a relative porosity index (*Ω*_t_ = 0–1) determined by the presence probability of each volume of veins with a cumulative number index of sealed crack (*ω*_t_ = 0–1), which is a rate between a cumulative number of completely sealed crack and total number of possible length-aperture width combinations obtained in this study (Supplementary Method). We assume that all cracks are opened (*Ω*_t_ = 1, *ω*_t_ = 0) at the time of earthquake rupture (*t* = 0) to show the range of duration of the porosity recovery period, and then *Ω*_t_ will decrease toward zero and *ω*_t_ will increase toward one by silica precipitation, respectively (Supplementary Method). This calculation does not consider the porosity of the host rock. Figure 6 shows that the relative crack porosity at Δ*P* = 25 MPa could recover by 89% (*Ω*_t_ = 0.1) to >99% (*Ω*_t_ < 0.01) within 9 × 10^1^–3 × 10^2^ years, respectively. The cumulative frequency of complete sealed crack number is 89% within 90 years (Fig. 6). At Δ*P* = 10 MPa, the relative porosity could recover by 38% (*Ω*_t_ = 0.62) to 66% (*Ω*_t_ = 0.34) within 9 × 10^1^–3 × 10^2^ years, respectively (Fig. 6), and 90% (*Ω*_t_ = 0.10) within 8 × 10^2^ years. The cumulative frequency of complete sealed crack number is 45% within 9 × 10^1^ years, which is almost half of that at Δ*P* = 25 MPa (Fig. 6).

## Discussion

The sealing time of a crack depends on various factors, including porosity and permeability in the host rock, and fluid composition. For the area around the Nobeoka Thrust, estimates of sealing time are more strongly influenced by the permeability of the host rocks than the porosity. An increase in host-rock porosity from 3% to 10% reduces the sealing time by ~30% (Fig. 4a), and an increase in permeability from 1 × 10^–18^ m^2^ to 1 × 10^–20^ m^2^ results in a longer sealing time from 6.6 × 10^–1^ to 6.6 × 10^1^ years (Fig. 4a). Several models of vein formation have been proposed, based on the diffusive transport of silica and pressure dissolution in the host rock^12,18^. These studies also describe porosity reduction around the fault, but their model considered smaller cracks and lower Δ*P* than in the present study. The dominant transport mechanism contributing to quartz vein formation is diffusion at lower Δ*P* and advection at higher Δ*P* (Fig. 4a). The Δ*P* value that marks the transition in transport mechanism becomes lower with increasing permeability and porosity (Fig. 4a). Accordingly, the conditions (*T*, *P*, Δ*P*, vein size, host rock properties) inferred for the extensional quartz veins around the Nobeoka Thrust indicate that advection was the dominant transport mechanism during quartz vein formation.

The salinity and pH of a fluid affect quartz solubility^34^ and the rate of quartz dissolution–precipitation^38^. For example, at Δ*P* = ~160 MPa, quartz solubility in seawater is ~1.6 × 10^1^ mg/kg(H_2_O) lower than in pure water^34^ (Supplementary Note) and the rate of quartz dissolution in seawater is about one order higher than that in pure water^38^. Therefore, the sealing time of extensional quartz veins that formed from fluids with compositions similar to those that contributed to vein formation around the Nobeoka Thrust (i.e., neutral pH^22^ and salinity similar to or less than seawater^27^) could be slightly shorter than that estimated in this study based on an assumption of pure water.

In natural systems, extensional quartz veins may occur in clusters, where the fluid catchments of neighboring cracks overlap with each other. Here we compare the sealing time of two simple systems, both of which contain the same volume of open cracks (1.0 × 10^–7^ m^2^). One system comprises a single crack (*w*_v_ = 1.0 × 10^2^ μm, *V*_crack_ = 1.0 × 10^–6^ m^2^) and the other a cluster of 100 thin cracks of the same size (*w*_v_ = 1.0 × 10^0^ μm, *V*_crack_ = 1.0 × 10^–8^ m^2^ for each crack). At Δ*P* = 25 MPa, the sealing time of the cluster (2.1 × 10^0^ years) is ~10^2^ times faster than that of the single crack (2.1 × 10^2^ years) because of the larger surface area for quartz precipitation (Supplementary Eq. 1). However, it is not always clear whether all veins in a cluster formed during the same event. Further analyses of natural occurrences of vein clusters and the development of models will be required to enable more accurate estimates of sealing time.

The recurrence intervals of subduction thrust earthquakes^39–42^ are generally shorter than those of inland earthquakes^43–46^. Since the Nobeoka Thrust is a good analogue of the seismogenic splay faults observed in the Nankai Trough^47^, we provide quantitative constraints to link silica precipitation with the recurrence of subduction thrust earthquakes in the Nankai Trough. At a depth of 10 km, comparable to the deeper part of the modern megasplay fault in the Nankai Trough^19,48^, the sealing time of the extensional cracks smaller than *w*_v_ = 5.2 × 10^1^ μm × *l*_v_ = 7.4 × 10^0^ cm (~5.6 × 10^1^ years; Fig. 4), is similar to the expected duration of the extensional stress regime in the Nankai Trough (~3 × 10^1^ years)^1,2^. For complete sealing of larger cracks, silica precipitation should continue in the openings of larger cracks even after the stress regime changes to compression, because our field studies revealed no evidence for multiple crack–seal events in any single extensional quartz vein (Fig. 2d). Considering that the rate of quartz overgrowth depends on the crystallographic orientations of seed crystals on crack walls, crack sealing in large open cavities is heterogeneous, and quartz crystals grow quickly from both sidewalls to impinge on one another^49^; thus, collapse of the cracks is prevented, even after a change in stress regime.

Under the conditions expected along the Nobeoka Thrust, almost all extensional quartz veins form within ~1 × 10^2^ years (Fig. 5), and the relative crack porosity at Δ*P* = 25 MPa could recover by >99% within ~3 × 10^2^ years (Fig. 6) known recurrence interval of megathrust events in the Nankai Trough, respectively^1,2^. This result suggests that when megaearthquakes form extensional cracks in the accretionary wedge, the porosity is nearly recovered by silica precipitation within the aseismic interval, regardless of crack size. Figure 6 also suggests that a larger pressure drop facilitates a much more drastic porosity recovery. A spherical reservoir with a radius of 7.5 × 10^–1^–1.0 × 10^0^ m, as required to seal a crack of mean size in the model of this study (Fig. 4b), is greater than the vein spacing observed in thin sections, suggesting that the set of quartz veins observed in our field study might not have formed from a single event. However, with the assumption that cracks with all possible combinations of length and aperture width are opened at the time of earthquake rupture (Fig. 6), our model is applicable to any number of crack openings, because the relative porosity is the ratio of the number of sealed cracks to the number of open cracks (Supplementary Method).

A decrease in sandstone porosity leads to the permeability decrease as recognized in various hydrostatic compression tests^50^, which may result in increased fluid pressure due to sealing of flow paths and supplying of fluid from deeper parts^22^, thereby leading to an increased probability of earthquake occurrence^36,51^. The fault-valve model^8^ describes a cycle wherein instantaneous permeability rises and fluid pressure drops at the time of an earthquake; these quantities then decrease and rise again, respectively, as the fault approaches the next earthquake. Therefore, the geochemical evolution of accretionary wedge porosity, as proposed in this study, relates to the evolution of permeability and fluid pressure in the earthquake cycle. Decreasing porosity enhances the build-up of pore fluid pressure because the fluid is supplied by flowing or percolating in the bulk and along faults or décollement during both pre- and post-seismic^27^. Pore pressure builds-up with increasing the number of closed extension cracks, which may not be in one-to-one relationship, and its timescale is similar to that of crack sealing. The high porosity generated at the time of an earthquake under extensional stress is followed by a rapid decrease just after the earthquake. During the low porosity regime, when the likelihood of the next earthquake is already high, porosity still decrease due to silica precipitation in larger cracks when the pressure difference between the host rock and cracks is maintained before an earthquake (Fig. 6). We conclude that silica precipitation generates subcritical conditions for earthquakes (i.e., lower porosity, lower permeability, higher fluid pressure) and thus controls earthquake recurrence intervals in seismogenic zones. The next megaearthquake may occur when these conditions reach critical values.

### Data availability

The datasets generated during the current study are available from the corresponding author on reasonable request.

## Electronic supplementary material

Supplementary information
