# Supplementary material for: Silica precipitation potentially controls earthquake recurrence in seismogenic zones
Source: Sci Rep. 2017 Oct 17;7:13337. doi: 10.1038/s41598-017-13597-5 (PMC5645327; doi:10.1038/s41598-017-13597-5)
Supplement: Supplementary file 1 — Supplementary information [file 41598_2017_13597_MOESM1_ESM.pdf]

Supplementary information for

Silica precipitation potentially controls earthquake recurrence in seismogenic zones
Hanae Saishu[1,2,*], Atsushi Okamoto[3,+], and Makoto Otsubo[2,+]

[1]Renewable Energy Research Center, National Institute of Advanced Industrial Science and Technology (AIST)
[2]Geological Survey of Japan, National Institute of Advanced Industrial Science and Technology (AIST)
[3]Graduate School of Environmental Studies, Tohoku University
[*]correspondence and requests for materials should be addressed to H.S. (e-mail: saishu.h@aist.go.jp)
[+]these authors contributed equally to this work

Calculation of sealing time using the model of crack in reservoir via advection and kinetic reactions

The sealing time of an extension crack by quartz precipitation on pre-existing quartz surfaces is estimated based on the kinetic equation for precipitation as follows[1]:

$$\frac{\partial C_{\mathrm{SiO2}}}{\partial t_{\mathrm{r}}} = k_{-} \frac{A_{\mathrm{Qtz}}}{M_{\mathrm{crack}}} (C_{\mathrm{SiO2,Qtz,eq}} - C_{\mathrm{SiO2}}) \,, \tag{2}$$

where t_r is the residence time of fluid in the crack. The SiO_2 concentration in the input fluid (C_{SiO2}) in mg/kg(H_2O) is the quartz solubility under the initial P–T conditions of the host rock. The value of $C_{SiO2,Qtz,eq}$ is the quartz solubility at fluid pressure in a crack (P_{crack}) after a fluid pressure drop (ΔP) as follows:

$$\Delta P = P_{\mathrm{host}} - P_{\mathrm{crack}}, \tag{3}$$

where P_{host} is fluid pressure in the host rock before a fluid pressure drop. The precipitation rate constant of quartz (k_-) was determined empirically as follows[2]:

$$\log k_{-} = -0.0886 - 2638/T, \tag{4}$$

where T is temperature in K. The model of an extension crack assumes a disk shape. The area of one crack wall (A_{crack}) in m^2 and the volume of the crack (V_{crack}) in m^3 are as follows:

$$A_{\mathrm{crack}} = \pi\, (l_{\mathrm{v}}/2)^2, \tag{5}$$
$$V_{\mathrm{crack}} = \pi\, w_{\mathrm{v}}\, (l_{\mathrm{v}}/2)^2, \tag{6}$$

where l_v and w_v are the length and width of a quartz vein as measured in field studies (e.g., Figs. 2a,b), which are assumed to be the diameter and height of a disk-shaped crack in our model, respectively. The area of quartz precipitation and the growth of quartz on crack walls (A_{Qtz}) in m^2 is computed as follows:

$$A_{\mathrm{Qtz}} = A_{\mathrm{crack}} \times \Psi_{\mathrm{Qtz}}, \tag{7}$$

where Ψ_{Qtz} is the proportion of quartz in the host rock. The mass of fluid in the crack (M_{crack}) in kg is determined as follows:

$$M_{\mathrm{crack}} = V_{\mathrm{crack}} \times V_{\mathrm{sp}}^{-1}, \tag{8}$$

where V_{sp} is the specific volume of water in m^3/kg at 250 °C and at the pressure achieved after the fluid pressure drop in m^3/kg.

At the beginning of the calculation, the apparent flow rate (Q_{ap}) in m^3/s is set to estimate the apparent residence time of fluid in the crack ($t_{r,ap}$) in s as follows:

$$t_{r,ap} = V_{crack} \times Q_{ap}^{-1}. \tag{9}$$

Replacement of the residence time (t_r) in equation (2) with $t_{r,ap}$ results in a change in SiO_2 concentration due to quartz precipitation in the crack (ΔC_{SiO2}) in mg/kg(H_2O) under the conditions of the apparent flow rate. The total volume of quartz precipitate in 1 kg of fluid (V_{Qtz}) in m^3/kg is

$$V_{Qtz} = \Delta C_{SiO2} / \varphi_{Qtz}, \tag{10}$$

where φ_{Qtz} is the density of quartz (2.65 g/cm^3). Thus, the sealing time of the whole crack volume (t_s) in s is

$$t_s = (V_{crack} / V_{Qtz}) \times V_{sp} \times Q_{ap}^{-1}, \tag{11}$$

and the total volume of fluid required to seal the whole crack ($V_{f,seal}$) in m^3, or equivalently, for the volume of silica precipitated to equal that of the crack, are estimated as follows:

$$V_{f,seal} = t_s \times Q_{ap} . \tag{12}$$

The volume of a spherical reservoir around the crack (V_{res}) in m^3 required to seal the crack volume ($V_{f,seal}$) in m^3 can be written as the following pair of functions:

$$V_{res} = V_{f,seal}/\phi, \tag{13}$$
$$V_{res} = 3/4\ \pi R^3, \tag{14}$$

where ϕ is the porosity of the host rock and R is the radius of the reservoir in m. As a result, R is determined using both equations (13) and (14). Thus, the flow rate into a crack (Q_D) in m^3/s can be determined from Darcy's law as follows:

$$\frac{Q_D}{A_{crack}} = -\frac{\kappa}{\mu}\frac{\Delta P}{R}, \tag{15}$$

where κ is the permeability of the host rock (sandstone), μ is the viscosity of the fluid, and ΔP in Pa is the pressure difference between the host rock and the crack, or equivalently, a fluid pressure drop in equation (3). Here, the apparent flow rate (Q_{ap}) used to determine R in equations (2)–(14) is not the same as the flow rate into a crack (Q_D) in Darcy's law in equation (15). Thus, we calculate the optimal flow rate (Q_k) in m^3/s in the loop calculation. First, Q_{ap} is set (small value) to calculate R and Q_D by using equations (2)–(15). Q_{ap} is compared with Q_D, and if Q_{ap} is lower than 99.9% of Q_D, Q_{ap} is increased in set step (e.g., $Q_{ap,i}$ = 0.1 m^3/s, step = 0.01 m^3/s, $Q_{ap,i+1}$ = 0.11 m^3/s). During loop calculation, when Q_{ap} = (or slightly higher than) $Q_D \times 0.999$, we set $Q_k = Q_{ap} = Q_D$, which results the optimal volume of fluid, crack sealing time and other parameters in the conditions.

Realistic conditions around the Nobeoka Thrust

Temperature and pressure in the host rock at 10 km depth are set to 250 °C and 260 MPa[3,4], respectively. The host rock of sandstone[3] is composed of 52% (Ψ_{Qtz} = 0.52) quartz[5]. The neutron porosity of the host rock (ϕ) falls in the range 3% to >10%, as recorded by geophysical wireline logs across the Nobeoka Thrust in a borehole experiment in 2011 by SRED and Raax Co., Ltd.[6]; we used 3% (ϕ = 0.03) in this work because fewer cracks existed before the earthquake. The permeability of the host rock (κ) falls within the range 1×10^{-20} to 1×10^{-18} m^2 as measured by a triaxial pressure apparatus[7]; we used an average value, $\kappa = 1.0 \times 10^{-19}$ m^2, in this study. The rate constant for silica precipitation via quartz growth on quartz surfaces (k_-) is 7.4×10^{-6} s^{-1} at 250 °C, following the Arrhenius relation of the experiment[2].

The SiO_2 concentration in the fluid in the host rock (C_{SiO2}) is 6.0×10^2 mg/kg(H_2O) as a result of pure water saturated via quartz at 250 °C and 260 MPa (Fig. 3)[8]. The pore fluid pressure in a crack is assumed to decrease close to the maximum hydrostatic pressure. The hydrostatic pressure at 10 km depth is 98 MPa. Therefore, the difference in pressure (ΔP) is ~160 MPa (Fig. 3). At 250 °C, silica precipitation occurs only in the liquid phase because boiling occurs at ≤4 MPa. The specific volume and viscosity of water is $V_{sp} = 1.1 \times 10^0$–1.2×10^0 cm^3/g[8] and $\mu = 1.5 \times 10^{-4}$–1.1×10^{-4} Pa[9], respectively, depending on ΔP = 0.01–160 MPa. When pore fluid pressure decreases to hydrostatic (ΔP = 160 MPa), the solubility of quartz in pure water decreases to 5.0×10^2 mg/kg(H_2O) (Fig. 3)[8]. Under these conditions, the quartz solubility in seawater (3 wt% NaCl) is 5.5×10^2 mg/kg(H_2O) and 4.8×10^2 mg/kg(H_2O) at lithostatic and near-hydrostatic pressure (ΔP = 160 MPa), respectively[8]. The maximum difference in quartz solubility in pure water, 9.4×10^1 mg/kg(H_2O), is larger than that in sea water, 7.8×10^1 mg/kg(H_2O). The quartz dissolution rate is strongly dependent on pH rather than salinity[10]. Fluid inclusion analysis in the Shimanto Belt of Kyushu, Japan revealed that typical fluid salinity is up to 5.5 wt% NaCl and is mainly similar to or lower than that of seawater[11]. The stability fields of the vein minerals indicate that extension veins formed from relatively oxidized, locally derived pore fluids of neutral pH[12], in the case of a dissolution rate in sea water of about one order higher than that in pure water[10]. Therefore, the sealing time of a crack would be similar to or shorter than that estimated in this study (Figs. 4–6 in the main manuscript).

Diffusion model

In the diffusion model of ref. 13, the sealing time of crack by diffusion (t_D) of a crack is written as follows:

$$t_{\mathrm{D}} = \frac{k_l l^2 + 2\bar{V}_{\mathrm{q}}\phi\tau D_f F_{\mathrm{d}}\hat{C}_2 l}{2k_l\bar{V}_{\mathrm{q}}\phi\tau D_f F_{\mathrm{d}}(\hat{C}_1 - \hat{C}_2)}, \quad (16)$$

where V_q is the molar volume of quartz (2.2×10^{-5} m^3/mol), ϕ is matrix porosity (porosity of the host rock, 3%), τ is tortuosity (1), D_f is the pore fluid diffusion coefficient (1×10^{-8} m^2/s), and F_d is the volume fraction of quartz dissolved out of the depletion zone (0.40; i.e., 40%). The length of the quartz fibre (l) in m is the same as the mode aperture width of the quartz vein ($w_v = 5.2 \times 10^1$ μm) in this study. The local equilibrium concentrations at the sites of dissolution and growth (C_1, C_2) in mol/m^3 are the same as the concentrations of SiO_2 in the fluid at the initial lithostatic pressure (C_{SiO2}) and after fluid pressure drop ($C_{SiO2,Qtz,eq}$) at 250 °C, respectively. Since the fibrous overgrowths by fluid diffusion are limited by

dissolution rather than precipitation[10], the linear dissolution rate constant (k_l) in m/s in equation (16), is determined by using molar volume of quartz (V_q), equilibrium constant and precipitation rate constant (k_-)[2], based on the quartz-water reaction[1]. The dissolution rate constant (k_l) is in range from 1.6×10^{-12} m/s to 1.1×10^{-12} m/s at ΔP from 0.1 MPa to 160 MPa, respectively.

Size variations in quartz veins and changes in the relative porosity index

The presence probability (or frequency) of extensional quartz veins along the Nobeoka Thrust ($\Phi_{n,m}$) is evaluated as follows:

$$\Phi_{n,m} = (x_n \times y_m)/100, \quad (17)$$

where x_n and y_m are the nth and mth relative frequency values of aperture width ($w_{v,n}$) and length ($l_{v,m}$), which are determined at each log-0.1 step in the ranges 1×10^1–1×10^3 μm and 1×10^0–1×10^2 cm in the histograms, respectively (Fig. 2a,b). In these classifications, the values of both n and m are 1–21. The total number of possible n–m combinations is 441 (21 × 21), 225 of which are considered presence-probable conditions ($\Phi_{n,m} > 0$).

To estimate the porosity change, we determined the relative porosity index (Ω_t) from 0 to 1 at time t_p in year. $\Omega_t = 0$ is just before an earthquake, where the porosity is the initial value of the host rock without any cracks; $\Omega_t = 1$ is just after earthquake ($t_p = 0$), when all cracks of presence-probable size have opened (Fig. 6). First, the volume of the disk-shaped crack (n, m) ($V_{crack,n,m}$) and its sealing time ($t_{s,n,m}$) are calculated in the model of this study. Next, the contribution ratio of the crack (n, m) to the total porosity ($\omega_{n,m}$) is weighted and normalized using the presence probability of the crack size as follows:

$$\omega_{n,m} = (V_{crack,n,m} \times \Phi_{n,m})/\sum\sum(V_{crack,n,m} \times \Phi_{n,m}). \quad (18)$$

Based on the assumption that a crack sealed in less time contributes to porosity healing occurring earlier, we sorted the crack sealing times (n, m) ($t_{s,n,m} = t_{s,i}$), where i is the sort index ranging from 1 to 225, corresponding to shorter to longer times, respectively. The relative contribution ratio of crack size to porosity was also sorted by sealing time, as $\omega_{n,m} = \omega_i$. Here, we assume that the precipitation rate is constant until a crack is sealed completely. As a result, the contribution ratio of the crack (n, m), rewritten as the crack sort index i on relative porosity Ω_i at time t_p, can be determined as follows:

$$\Omega_i = \omega_i, \text{ when } t_p \geq t_{s,i}, \quad (19)$$
$$\Omega_i = \omega_i, \times (t/t_{s,i}), \text{ when } t_p < t_{s,i}. \quad (20)$$

The sealed crack index at time t_p is the maximum value of i that satisfies equation (19), and the total change in relative porosity index at time t_p is estimated as follows:

$$\Omega_t = \Omega_0 - \sum\Omega_i, \quad (21)$$

where $\Omega_0 = 1$ ($t_p = 0$) when all cracks of presence-probable size open. In addition, the cumulative number index of sealed crack ($\omega_t = 0$–1) is a rate between a cumulative number of cracks completely sealed (i.e., satisfy the conditions of equation (19)) and total number of possible length-aperture width combinations (225).

References

1. Rimstidt, J. D. & Barnes, H. L. The kinetics of silica–water reactions. *Geochim. Cosmochim. Acta* **44**, 1683–1699 (1980).
2. Okamoto, A., Saishu, H., Hirano, N. & Tsuchiya, N. Mineralogical and textural variation of silica minerals in hydrothermal flow-through experiments: Implications for quartz vein formation. *Geochim. Cosmochim. Acta* **74**, 3692–3706 (2010).
3. Kondo, H. *et al*. Deformation and fluid flow of a major out-of-sequence thrust located at seismogenic depth in an accretionary complex: Nobeoka Thrust in the Shimanto Belt, Kyushu, Japan. *Tectonics* **24**, 1–16 (2005).
4. Otsubo, M. *et al*. Variation of stress and pore fluid pressure using vein orientation along seismogenic megasplay fault - example of Nobeoka Thrust, southwestern Japan. *Island Arc* **25**, 421–432 (2016).
5. Okamoto, A. & Sekine, K. Textures of syntaxial quartz veins synthesized by hydrothermal experiments. *J. Struct. Geol.* **33**, 1764–1775 (2011).
6. Hamahashi, M. *et al*. Contrasts in physical properties between the hanging wall and footwall of an exhumed seismogenic megasplay fault in a subduction zone - An example from the Nobeoka Thrust Drilling Project. *Geochem. Geophys. Geosyst.* **14**, 5354–70 (2013).
7. Kato, A. *et al*. Permeability structure around an ancient exhumed subduction-zone fault. *Geophys. Res. Lett.* **31**, L06602 (2004).
8. Akinfiev, N. N. & Diamond, L. W. A simple predictive model of quartz solubility in water-salt-CO2 systems at temperatures up to 1000 °C and pressures up to 1000 MPa. *Geochim. Cosmochim. Acta* **73**, 1597–1608 (2009).
9. Parry, W. T. *ASME International Steam Tables for Industrial Use* (ASME Press. New York, 2000).
10. Dove, P.M. The dissolution kinetics of quartz in sodium chloride solutions at 25 °C to 300 °C. *Am. J. Sci.* **294**, 665–712 (1994).
11. Raimbourg, H. *et al.* Fluid circulation in the depths of accretionary prisms: an example of the Shimanto Belt, Kyushu, Japan. *Techtonophys.* **655**, 161–176 (2015).
12. Yamaguchi, A., Cox, S. F., Kimura, G. & Okamoto, S. Dynamic changes in fluid redox state associated with episodic fault rupture along a megasplay fault in a subduction zone. *Earth Planet. Sci. Lett.* **302**, 369–377 (2011).
13. Fisher, D. M. & Brantley, S. L. Model of quartz overgrowth and vein formation: deformation and episodic fluid flow in an ancient subduction zone. *J. Geophys. Res.* **97**, 20043–20061 (1992).
